# Validation of Risk Prediction Models to Inform Clinical Decisions After Acute Kidney Injury

**DOI:** 10.1053/j.ajkd.2020.12.008

**Published:** 2021-07

**Authors:** Simon Sawhney, Zhi Tan, Corri Black, Angharad Marks, David J. Mclernon, Paul Ronksley, Matthew T. James

**Affiliations:** 1 Aberdeen Centre for Health Data Sciences, University of Aberdeen, Aberdeen, Scotland; 2 National Health Service Grampian, Aberdeen, Scotland; 3 Cumming School of Medicine, University of Calgary, Calgary, Alberta, Canada; 4 Renal Unit, Abertawe Bro Morgannwg University Health Board, Swansea, Wales

**Keywords:** acute kidney injury (AKI), chronic kidney disease (CKD), CKD progression, CKD surveillance, death, follow-up care, hospital readmission, model-guided decisions, mortality, net benefit, post-AKI care, post-discharge monitoring, risk prediction

## Abstract

**Rationale & Objective:**

There is limited evidence to guide follow-up after acute kidney injury (AKI). Knowledge gaps include which patients to prioritize, at what time point, and for mitigation of which outcomes. In this study, we sought to compare the net benefit of risk model–based clinical decisions following AKI.

**Study Design:**

External validation of 2 risk models of AKI outcomes: the Grampian -Aberdeen (United Kingdom) AKI readmissions model and the Alberta (Canada) kidney disease risk model of chronic kidney disease (CKD) glomerular (G) filtration rate categories 4 and 5 (CKD G4 and G5). Process mining to delineate existing care pathways.

**Setting & Participants:**

Validation was based on data from adult hospital survivors of AKI from Grampian, 2011-2013.

**Predictors:**

KDIGO-based measures of AKI severity and comorbidities specified in the original models.

**Outcomes:**

Death or readmission within 90 days for all hospital survivors. Progression to new CKD G4-G5 for patients surviving at least 90 days after AKI.

**Analytical Approach:**

Decision curve analysis to assess the “net benefit” of use of risk models to guide clinical care compared to alternative approaches (eg, prioritizing all AKI, severe AKI, or only those without kidney recovery).

**Results:**

26,575 of 105,461 hospital survivors in Grampian (mean age, 60.9 ± 19.8 [SD] years) were included for validation of the death or readmission model, and 9,382 patients (mean age, 60.9 ± 19.8 years) for the CKD G4-G5 model. Both models discriminated well (area under the curve [AUC], 0.77 and 0.86, respectively). Decision curve analysis showed greater net benefit for follow up of all AKI than only severe AKI in most cases. Both original and refitted models provided net benefit superior to any other decision strategy. In process mining of all hospital discharges, 41% of readmissions and deaths occurred among people recovering after AKI. 1,464 of 3,776 people (39%) readmitted after AKI had received no intervening monitoring.

**Limitations:**

Both original models overstated risks, indicating a need for regular updating.

**Conclusions:**

Follow up after AKI has potential net benefit for preempting readmissions, death, and subsequent CKD progression. Decisions could be improved by using risk models and by focusing on AKI across a full spectrum of severity. The current lack of monitoring among many with poor outcomes indicates possible opportunities for implementation of decision support.

**Plain-Language Summary**

Despite frequent poor outcomes, there is limited evidence to guide the way in which we prioritize care after acute kidney injury (AKI). This study validates 2 clinical risk models for outcomes in hospital survivors and AKI survivors. We used decision curve analysis to compare which decision strategies provide more benefit than harm. We found that risk models predicting death or readmission and chronic kidney disease have the potential to assist follow-up decisions after AKI and could be superior to alternative strategies such as prioritizing AKI severity or kidney recovery alone. We also found that many patients currently receive little or no postdischarge monitoring after AKI. This indicates possible opportunities for the implementation of decision support to guide postdischarge care for people hospitalized with AKI.

Editorial, p. 16

Among the 1 in 7 people in hospital who develop acute kidney injury (AKI),^1^ many continue to experience poor health outcomes even after discharge, including a 1 in 3 risk of unplanned readmissions within 90 days (especially with heart failure),^2^, ^3^, ^4^, ^5^ and incomplete recovery of kidney function leading to new or progressive chronic kidney disease (CKD) over the first year.^6^^,^^7^ From this large and heterogeneous group of people with AKI, it remains unclear which individuals should receive follow up after AKI and for what reasons.

Current international guidelines recommend follow up of all people with AKI after 90 days.^8^ Post-AKI return clinics have been incorporated into some health systems, but a single approach may not be appropriate or efficient for all patients.^9^^,^^10^ Thus, for some, an early period of heightened surveillance and care after AKI could be beneficial; but for others, additional monitoring and follow-up interventions may add little overall benefit to their health and may even introduce unnecessary costs.^11^

Risk prediction models can be used to assist complex decision making. Decision models combine available information to help health professionals quantify an individual’s risk of adverse outcomes. Two recent risk models have been developed to predict outcomes of AKI after hospital discharge.^2^^,^^6^ The Aberdeen (United Kingdom) risk tool predicts death or hospital readmission for all people (with and without AKI) in the early postdischarge period. The model, which includes AKI as a key predictor, could be used by nonspecialists to target people who may benefit from early surveillance (eg, in primary care) to preempt or prevent recurrent acute illness.^2^ A complementary focused risk prediction model from Alberta (Canada) has been developed to predict the risk of progression to new CKD glomerular filtration rate categories 4 and 5 (G4-G5) among survivors of AKI and could be used to identify individuals who may benefit from referral to a nephrologist for specialized kidney care.^6^ To be of value, these risk models must (1) distinguish well between high- and low-risk people; (2) provide accurate absolute risk predictions; and (3) lead to decisions that will do more good than harm compared with alternative decision strategies. This final attribute is known as “net benefit.” Although statistical metrics that show whether a model provides accurate predictions are frequently reported, the clinical usefulness of the model or the “net benefit” of model-based clinical decisions over alternative approaches is rarely reported^12^, ^13^, ^14^, ^15^, ^16^, ^17^ and is the focus of this study.

In this study, we formally evaluated the statistical performance and net benefit of these 2 complementary risk models relevant to follow-up care after AKI, validating them using new datasets. First, to support nonspecialists, we used the Aberdeen risk model to examine whether, from among all hospital admissions (with or without AKI), the targeted follow-up of those people who have had AKI (according to clinical guidelines) would have positive net benefit to preempt early deaths and readmissions, whether net benefit would be improved by using a risk model, and whether net benefit could be improved by targeting only those with the most severe AKI episodes. Next, to support prioritization of specialist kidney care, we applied the Alberta risk model to AKI survivors to assess whether the net benefit for targeting kidney care follow-up would be greatest by using the risk model, by targeting severe AKI, or by targeting those without recovery.

Finally, to characterize the extent of existing post-AKI care and the potential clinical need for decision support, we examined the care processes and monitoring of people with AKI discharged from hospital over a 4-year period.

## Methods

We reported this prediction model validation study according to the TRIPOD statement^14^. Permissions for this study were provided by North of Scotland Research Ethics Committee (18/NS/0051), Grampian Caldicott guardian, and NHS Research and Development. Routinely collected health data were de-identified and analyzed within a secure environment, and therefore permissions were provided without a requirement for informed consent to be obtained from the participants.

### Populations: Original Models

The Aberdeen AKI readmissions model was derived from people hospitalized in 2003 in Grampian, North Scotland, using the Grampian Laboratory Outcomes Morbidity and Mortality Study (GLOMMS).^2^ The derivation cohort included people who survived a hospitalization with or without AKI (n = 16,453; 3,065 outcomes; internal validation cohort C statistic, 0.70). All people with baseline estimated glomerular filtration rate (eGFR) <60 mL/min/1.73 m^2^ were included and a 20% random sample of those with normal baseline kidney function. People with a kidney transplant or receiving maintenance dialysis were excluded.

The Alberta model was derived using the Alberta Kidney Disease Network Database from all 3.5 million residents in the province, 2004-2014.^6^ The derivation population included adult residents of Alberta with a baseline eGFR >45 ml/min1.73m^2^ who survived at least 90 days after hospitalization with AKI (n = 9,973; 272 outcomes; C statistic of 0.87 in internal validation and of 0.81 in external validation cohort). People with a kidney transplant or receiving maintenance dialysis were excluded.

### Outcomes: Original Models

The Aberdeen readmission model predicts the risk of mortality or unplanned readmission within 90 days of discharge after a hospitalization. The purpose of the model was framed to support nonspecialists with discharge planning, with a risk threshold of 30% for targeting high-risk people.

The Alberta CKD G4-G5 model predicts the risk of progression to CKD G4-G5 within 1 year after hospitalization with AKI. A minimum of 2 outpatient assessments of eGFR < 30 mL/min/1.73 m^2^ separated by at least 3 months was required to meet the eGFR component of the definition. Estimation of GFR used the Chronic Kidney Disease Epidemiology Collaboration (CKD-EPI)^18^ equation. Risk thresholds of <1% and >10% have been proposed to guide follow-up decisions after AKI, including those who may benefit from specialist nephrology follow up (ClinicalTrials.gov identifier NCT02915575).

### Predictors: Original Models

In both development cohorts, AKI was determined and staged for severity using KDIGO-based AKI criteria. The Aberdeen readmissions model established AKI using a previously published and validated KDIGO-based algorithm. Briefly AKI was present if there was (1) a creatinine increase of >0.3 mg/dL within 48 hours; or (2) a 50% rise from the lowest creatinine in the past 7 days; or (3) a 50% rise from the median creatinine in the past 8-90 days, or 91-365 days if no closer samples existed.^1^^,^^19^ The Alberta CKD G4-G5 model established AKI based on a rise in creatinine during hospitalization of >0.3 mg/dL or >50% of their most recent outpatient prehospital baseline 7-365 days before admission.^6^

As previously described additional laboratory-derived variables included kidney function at baseline and discharge (creatinine and albuminuria within categories in the Alberta CKD G4-G5 model, and eGFR in Aberdeen readmissions model), admission circumstances (emergency admission, admission from residential care, rural home location) and comorbidities based on validated algorithms using International Classification of Diseases 10th revision (ICD-10) codes.^20^^,^^21^

### Analysis: Original Models

Both risk models were developed using multivariable logistic regression with stepwise backward variable selection and bootstrapping to identify consistent predictors included in the final model.^16^

### Validation Cohorts

#### Participants

Both models were validated in a separate cohort. To maximize reproducibility, analysis code from the original development of both models was shared to ensure that the cohort formation, outcomes, and predictors were defined consistently.

The Aberdeen readmissions model was temporally validated in a cohort of all adult Grampian residents admitted to hospital in Grampian in 2012 (with or without AKI). The Alberta CKD G4-G5 model was geographically validated using all adult Grampian residents admitted to hospital with AKI between 2011-2013 who had a baseline eGFR >45 mL/min1.73 m^2^ and survived at least 90 days after hospitalization.

### Statistical Analyses

#### Discrimination and Calibration

For both models the full logistic regression equation (all regression coefficients and model intercept) was used to calculate the predicted risks for each person in the external validation cohorts. Model discrimination (the ability to estimate higher risks for patients with the outcome and lower risks for those without the outcome) was compared based on the C statistic. Model calibration (how well estimated probabilities agree with observed outcomes) was assessed using calibration-in-the-large and the calibration slope. Calibration-in-the-large is the difference between the mean observed risk and the mean predicted risk and is a measure of systematic overprediction (negative) or underprediction (positive). The calibration slope is a measure of model fit derived by comparing predicted probabilities versus observed outcomes against a theoretic line for perfect calibration in which a perfect intercept equals 0 and a perfect slope has a gradient of 1. Calibration was visualized by plotting the observed proportions of outcomes versus mean predicted probability within tenths of increasing predicted risk.^16^^,^^17^^,^^22^

We determined a priori to perform model recalibration and refitting if there was any evidence of miscalibration (slope not equal to 1 or intercept not equal to 0). We recalibrated using logistic calibration^23^^,^^24^ and fitted a logistic regression model with the prognostic index (defined as the weighted sum of the predictor values in the new dataset, where the weights are the coefficients of the original models) as the only covariate. We then used the parameter estimate of the prognostic index (calibration slope) and the intercept (calibration intercept) to recalibrate the regression coefficients and the baseline risk of the original model^23^. To refit, we used only the variables that were in the original model and parameterized continuous variables in the same way as the original models. This was to avoid performance gains simply by saturating the refitted model with additional variables.

#### Decision Curve Analysis

Decision curve analysis is a plot of the “net benefit” against “threshold probabilities”, assessing the clinical usefulness of different models at appropriate thresholds for clinical use. It is described in detail elsewhere,^13^^,^^25^ and summarized briefly here. Net benefit measures the trade-off between true positives and false positives in a prediction model at different threshold probabilities. It is a sum of true-positive minus false-positive predictions weighted by the threshold probability as described in the following equation^25^:

$$\text{Net benefit}=\left(\frac{\text{true positive}}{\text{total sample size}}\right)-\left[\left(\frac{\text{false positive}}{\text{total sample size}}\right)\left(\frac{\text{threshold probability}}{1-\text{threshold probability}}\right)\right]$$

The threshold is a clinically derived value that varies depending on how risk averse the clinician is. It is a value where the clinician would be satisfied with the tradeoff between the harm (eg, death, readmission, or CKD progression) of delaying intervention (targeted follow-up) and unnecessary intervention. In AKI, a clinical trial chose a threshold of 0.1 for nephrology specialists’ follow up for CKD G4-G5 (ClinicalTrials.gov identifier NCT02915575). A threshold of 0.1 implies that for every 1 true-positive case identified by the decision strategy, fewer than 9 false positives would be an acceptable tradeoff. This means weighting the finding a high-risk patient as 9 times more important than avoiding unnecessary follow-up. Although the absolute value of net benefit is an abstract concept, the higher the positive value of net benefit, the greater the clinical value, allowing a comparison of different strategies for clinical decision making.

We used decision curve analysis to compare the original models, recalibrated models, refitted models, and also strategies of targeting postdischarge care to all people leaving the hospital with AKI, to all with AKI of stages 2 or 3, to all with a discharge eGFR <30 mL/min/1.73 m^2^, to all in the cohort (regardless of AKI), and to no people in the cohort. Recovery of kidney function after AKI may be uncertain and incomplete at hospital discharge, but we included discharge eGFR as a pragmatic decision strategy that clinicians may consider using as a proxy for persistent low GFR after AKI.

At the prespecified thresholds (1% and 10% for the Alberta CKD progression model, and 30% for the Aberdeen readmission model), we also reported classification of people by decision strategy and outcome in 2 × 2 tables, with the percentage correctly classified and net benefit. These analyses were performed using Stata version SE 16 software (StataCorp).

#### Process Mining

Process mining is a method for describing the organization, order, and timing within an event log. We used process mining software (DISCO, Fluxion 2020) to visualize the most common pathways for health care monitoring events of people leaving the hospital after AKI, and their subsequent routes back into hospital within 30 and 90 days. We linked kidney function monitoring events with paths depicted by arrows weighted by the number of people who moved between events, from hospital discharge to outpatient specialist clinics, primary care general practice, or accident and emergency, and ending at hospital readmission or the end of follow up (30 and 90 days in 2 respective analyses). We reported median time between events for each path. We used data from people with AKI who survived an acute hospital admission in Grampian during 2011-2014.

## Results

### Participants

The validation cohort for the Aberdeen readmissions model included 26,575 people and 2,927 events. The validation cohort of the Alberta CKD G4-G5 model included 9,382 people and 140 events.

Characteristics across all cohorts are reported in Table 1, Table 2. For the Aberdeen readmissions model, the validation cohort of all admissions in 2012 was larger than the original derivation cohort sample of those admitted in 2003. The validation cohort was younger and had a higher mean baseline eGFR. For the Alberta CKD G4-G5 model, the validation cohort and Alberta derivation cohorts were similar, but the validation cohort had a higher proportion of people with unmeasured albuminuria.

**Table 1:** Characteristics of Derivation and Validation Cohorts for Readmissions Model

	Derivation: Grampian 2003 (n = 16,453)	External Validation: Grampian 2012 (n = 26,575)
Demographics		
Age, y	66.1 ± 18.1	60.9 ± 19.8
Residential care	748 (4.5%)	413 (1.6%)
Rural home location	4,455 (27.1%)	7,646 (28.8%)
Admissions in past year		
No admissions	12,357 (75.1%)	13,897 (52.3%)
1 admission	2,465 (15.0%)	6,380 (24.0%)
2 admissions	864 (5.3%)	3,057 (11.5%)
3+ admissions	767 (4.7%)	3,241 (12.2%
Emergency admission	10,086 (61.3%)	18,940 (71.3%)
AKI status		
No AKI	13,830 (84.1%)	23,255 (87.5%)
AKI stage 1	1,718 (10.4%)	2,299 (8.7%)
AKI stage 2	574 (3.5%)	611 (2.3%)
AKI stage 3	331 (2.0%)	410 (1.5%)
Baseline eGFR, mL/min/1.73 m^2^	69.3 ± 25.2	86.3 ± 26.4
Comorbidities		
Cancer	1,383 (8.4%)	3,733 (14.0%)
Cardiac failure	909 (5.5%)	2,149 (8.1%)
Diabetes	1,112 (6.8%)	3,467 (13.0%)
Pulmonary	1,050 (6.4%)	4,990 (18.8%)

Values for continuous variables given as mean ± standard devaition; for categorial variables, as count (%). Abbreviations: AKI, acute kidney injury; eGFR, estimated glomerular filtration rate.

**Table 2:** Characteristics of Derivation and Validation Cohorts for CKD G4-G5 Model

	Derivation: Alberta (n = 9973)	Validation: Alberta (n = 4,985)	External Validation: Grampian 2011-2013 (n = 9,382)
Demographics			
Age, y	65.7 ± 14.9	66 ± 15.0	67.2 ± 15.4
Female sex	4,258 (42.7%)	2,091 (41.9%)	4,297 (45.8%)
Laboratory results			
Serum creatinine, mg/dL	1.0 ± 0.2	1.0 ± 0.2	0.9 ± 0.3
Baseline eGFR, mL/min/1.73 m^2^	76 ± 20.0	76 ± 20.0	77 ± 23.0
Albuminuria			
Normal	3,775 (37.8%)	1,881 (37.7%)	1,116 (11.9%)
Mild	1,373 (13.8%)	662 (13.3%)	598 (6.4%)
Heavy	494 (4.9%)	243 (4.9%)	321 (3.4%)
Unmeasured	4,331 (43.4%)	2,199 (44.1%)	7,347 (78.3%)
AKI status			
AKI stage 1	7,686 (77.1%)	3,806 (76.4%)	7,361 (78.5%)
AKI stage 2	1,357 (13.6%)	699 (14.0%)	1,239 (13.2%)
AKI stage 3	930 (9.3%)	480 (9.6%)	782 (8.3%)
Discharge serum creatinine			
<1.0 mg/dL	3,394 (34.0%)	1,721 (34.5%)	4,488 (47.8%)
1.0-<1.3 mg/dL	3,207 (32.2%)	1,542 (30.9%)	2,651 (28.3%)
1.3-<1.6 mg/dL	1,963 (19.7%)	1,012 (20.3%)	1,438 (15.3%)
1.6-<1.9 mg/dL	799 (8.0%)	383 (7.7%)	470 (5.0%)
>1.9 mg/dL	610 (6.1%)	327 (6.6%)	335 (3.6%)
Discharge eGFR, mL/min/1.73 m^2^	62.0 ± 23.0	62.0 ± 23.0	69.1 ± 25.0
Comorbidities			
Diabetes	920 (9.2%)	498 (10.0%)	2,093 (22.3%)
Cancer	2,638 (26.4%)	1,329 (26.7%)	1,918 (20.4%)
Heart failure	2,422 (24.3%)	1,197 (24.0%)	1,354 (14.4%)
Metastatic cancer	451 (4.5%)	221 (4.4%)	410 (4.4%)
Myocardial infarction	1,796 (18.0%)	902 (18.1%)	1,531 (16.3%)
Mild liver disease	302 (4.7%)	150 (4.7%)	357 (3.8%)
Moderate to severe liver disease	189 (1.9%)	99 (2.0%)	180 (1.9%)
Peripheral vascular disease	1,079 (10.8%)	505 (10.1%)	931 (9.9%)
Rheumatic	404 (4.0%)	215 (4.3%)	414 (4.4%)
Hypertension	1,734 (17.4%)	861 (17.3%)	4,571 (48.7%)
Procedures			
Cardiac catheterization	629 (6.3%)	316 (6.3%)	127 (1.4%)
Cardiac surgery	754 (7.6%)	301 (6.0%)	313 (3.3%)
Aortic aneurysm repair	139 (1.4%)	53 (1.1%)	43 (0.5%)
Ventilation^a^	1,752 (17.6%)	818 (16.4%)	147 (1.6%)
Dialysis	175 (1.8%)	83 (1.7%)	103 (1.1%)
Hospital LOS, d	9 [5-18]	9 [5-19]	9 [4-19]

Values for continuous variables given as mean ± standard deviation or median [interquartile range]; for categorical variables, as count (%). Abbreviations: AKI, acute kidney injury; CKD G4-G5, chronic kidney disease glomerular filtration rate categories 4 and 5; eGFR, estimated glomerular filtration rate; LOS, length of stay.

a Ventilation definition in Alberta dataset include mechanical ventilation, noninvasive ventilation and oxygen therapy. The Grampian dataset does not include oxygen therapy.

### Model Performance and Updating

**Figure 1:**
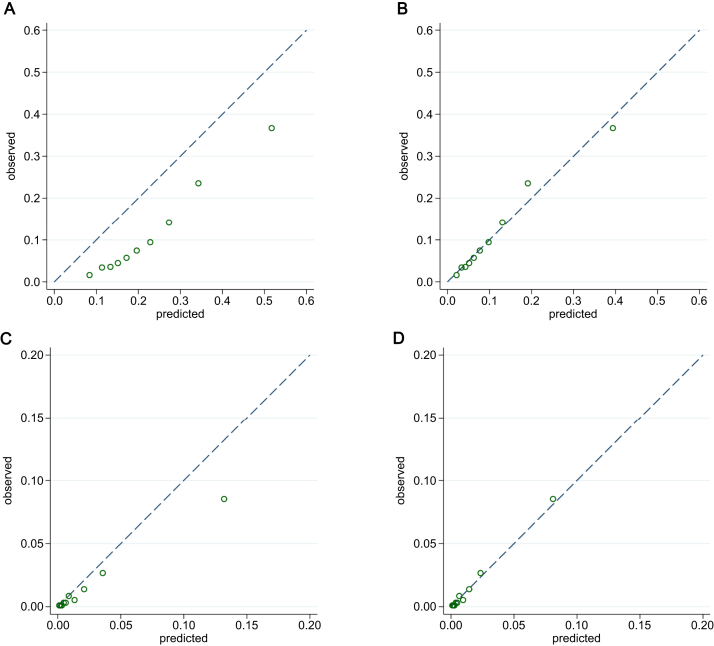
Calibration of (A) the reported Aberdeen readmissions model, and (B) after recalibration, and of (C) the reported Alberta chronic kidney disease glomerular filtration rate categories 4 and 5 model, and (D) after recalibration. The dashed line represents the line of perfect agreement; circles represent tenths of increasing predicted risk.

Fig 1 shows calibration plots for external validation of the original Aberdeen model for death or unplanned readmissions (Fig 1A), after recalibration of the slope and intercept (Fig 1B); and the original Alberta CKD G4-G5 model (Fig 1C), after recalibration of slope and intercept (Fig 1D). As shown in Table 3, both models discriminated well in the external validation cohorts (AUC of 0.77 for the death or readmissions model, and AUC of 0.86 for the CKD G4-G5 model). Both models overpredicted risks, which improved following recalibration. Table 4, Table 5 report the odds ratios and intercepts of the original and refitted Aberdeen death or readmissions model (Table 4) and refit Alberta CKD G4-G5 model (Table 5). The odds ratios were similar for refitted models except for albuminuria in the CKD G4-G5 model, for which an unmeasured value of albuminuria had a protective effect in the external validation cohort.

**Table 3:** Prediction Model Performance

	Aberdeen Readmissions Model		Alberta CKD G4-G5 Model	
	Original	Refitted	Original	Refitted
AUC (95% confidence interval)	0.773 (0.765-0.783)	0.786 (0.777-0.795)	0.855 (0.824-0.886)	0.866 (0.837-0.895)
Brier score	0.098	0.086	0.015	0.014
Predicted-to-observed ratio	2.042	1.000	1.531	1.001
Calibration intercept^a^	−0.928	0.000	−0.484	−0.001
Calibration slope	1.374	1.000	0.901	1.000

Abbreviations: AUC, area under the receiver operated curve; CKD G4-G5, chronic kidney disease glomerular filtration rate categories 4 and 5.

a Calibration-in-the-large.

**Table 4:** Original and Refitted Aberdeen Readmissions Model

	Original Derivation Model: Grampian 2003	Refitted Validation Model: Grampian 2012
Admission details		
Age, per year older	1.17 (1.13-1.21)	1.29 (1.25-1.34)
Residential care	1.37 (1.42-1.98)	2.46 (1.95-3.10)
Rural home location	0.86 (0.78-0.94)	0.95 (0.87-1.05)
Admissions in previous year, per admission	1.23 (1.18-1.27)	1.26 (1.23-1.29)
Emergency admission	1.89 (1.72-2.08)	2.2 (1.97-2.45)
AKI status		
No AKI	1.00 (reference)	1.00 (reference)
AKI stage 1	1.50 (1.33-1.70)	3.43 (3.08-3.82)
AKI stage 2	2.23 (1.85-2.68)	5.63 (4.70-6.75)
AKI stage 3	2.80 (2.22-3.53)	5.04 (4.05-6.27)
Baseline eGFR		
Linear term^a^	0.87 (0.80-0.94)	0.65 (0.61-0.70)
Square term^a^	1.01 (1.01-1.02)	1.03 (1.03-1.04)
Comorbidities		
Cancer	1.59 (1.37-1.82)	1.9 (1.71-2.12)
Cardiac failure	1.42 (1.21-1.66)	1.18 (1.04-1.34)
Diabetes	1.38 (1.19-1.60)	1.21 (1.09-1.35)
Pulmonary	1.47 (1.27-1.70)	1.46 (1.32-1.61)
Intercept for linear predictor	−1.876	−2.196

Unless otherwise indicated, values are odds ratio (95% confidence interval) for readmission or death within 90 days for all hospital survivors. Abbreviations: AKI, acute kidney injury; eGFR, estimated glomerular filtration rate.

a Baseline eGFR modelled per 10 mL/min/1.73 m^2^ increase with a combination of linear and quadratic terms.

**Table 5:** Original and Refitted Alberta CKD G4-G5 Model

	Original Derivation Model (Alberta)	Refitted Validation Model (Grampian 2011-2013)
Admission details		
Age, per year older	1.02 (1.01-1.03)	1.02 (1.01-1.04)
Female sex	2.93 (2.16-3.97)	3.05 (2.04-4.56)
AKI stage		
1	1.00 (reference)	1.00 (reference)
2	1.28 (0.88-1.87)	2.15 (1.33-3.47)
3	2.47 (1.73-3.52)	2.82 (1.70-4.67)
Baseline Scr, per 0.1 mg/dL greater	1.18 (1.10-1.18)	1.21 (1.14-1.30)
Discharge Scr		
<0.1 mg/dL	1.00 (reference)	1.00 (reference)
1.0-<1.3 mg/dL	2.93 (1.55-5.56)	4.1 (2.04-8.24)
1.3-<1.6 mg/dL	7.78 (4.19-14.44)	7.92 (3.91-16.02)
1.6-<1.9 mg/dL	11.35 (5.86-21.97)	11.31 (5.12-25.00)
>1.9 mg/dL	37.01 (19.46-70.37)	21.74 (10.10-46.75)
Albuminuria		
Normal	1.00 (reference)	1.00 (reference)
Mild	1.25 (0.81-1.92)	0.95 (0.47-1.93)
Heavy	3.13 (2.00-4.91)	2.00 (1.03-3.89)
Unmeasured	1.67 (1.21-2.29)	0.78 (0.47-1.29)
Intercept for linear predictor	−9.246	−8.755

Unless otherwise indicated, values are odds ratio (95% confidence interval) for progression to new CKD G4-G5, for patients surviving at least 90 days after AKI. Abbreviations: AKI, acute kidney injury; CKD G4-G5, chronic kidney disease glomerular filtration rate categories 4 and 5; Scr, serum creatinine.

### Net Benefit and Decision Curve Analysis

**Figure 2:**
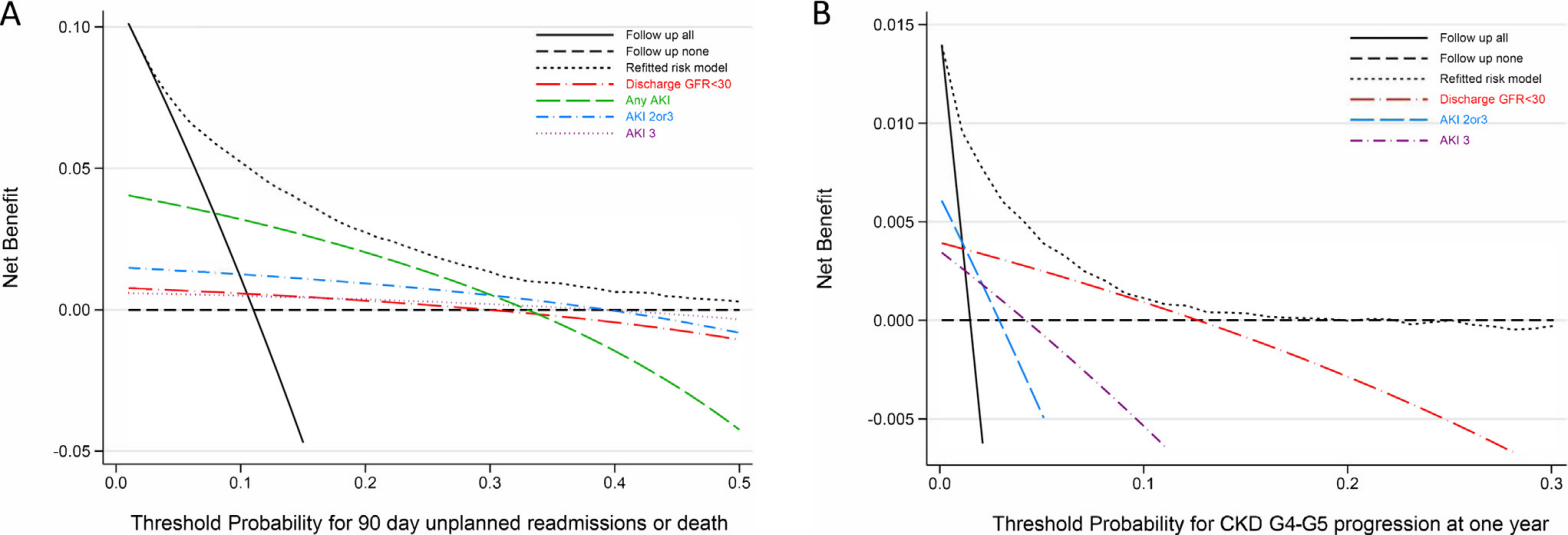
Decision curve analysis for the net benefit of the (A) Aberdeen readmissions model and (B) Alberta CKD G4-G5 model compared with alternative decision strategies. Net benefit represents the trade-off between true positives and false positives, with false positives weighted by the threshold probability from 0 (no penalization for false positives) to 1 (infinite penalization for false positives). At a threshold of 0.1, 1 true positive would be balanced by 9 false positives. Abbreviations: AKI, acute kidney injury; CKD G4-G5, chronic kidney disease glomerular filtration rate categories 4 and 5; GFR, glomerular filtration rate.

Figure 2 shows the results of decision curve analysis for both the Aberdeen death or readmissions model for all hospital survivors (Fig 2A) and the Alberta CKD G4-G5 model for AKI survivors (Fig 2B). For predicting death or readmission among all hospital survivors, follow-up of those with AKI was of positive net benefit, but a model-guided approach led to the greatest net benefit at the relevant risk thresholds. Fig S1 shows decision curve analysis of the net benefit for predicting death or readmission where the analysis has been restricted only to people with AKI. Again, the analysis shows that the greatest net benefit occurs by following the risk model, with limited benefit or potential net harm from following only people with severe AKI. For predicting CKD G4-G5 progression at 1 year among AKI survivors (Fig 2B), a model-guided approach had a small gain in net benefit that remained superior to a strategy guided by discharge eGFR <30 mL/min/1.73m^2^ at the prespecified 10% risk threshold. Tables S1 and S2 provide net benefit calculations at the 30% risk threshold for the Aberdeen readmissions model, and at 1% and 10% risk thresholds for the Alberta model. The superiority of a model-guided approach was consistent for both models regardless of whether the original, recalibrated, or refitted model was used.

### Process Mining

**Figure 3:**
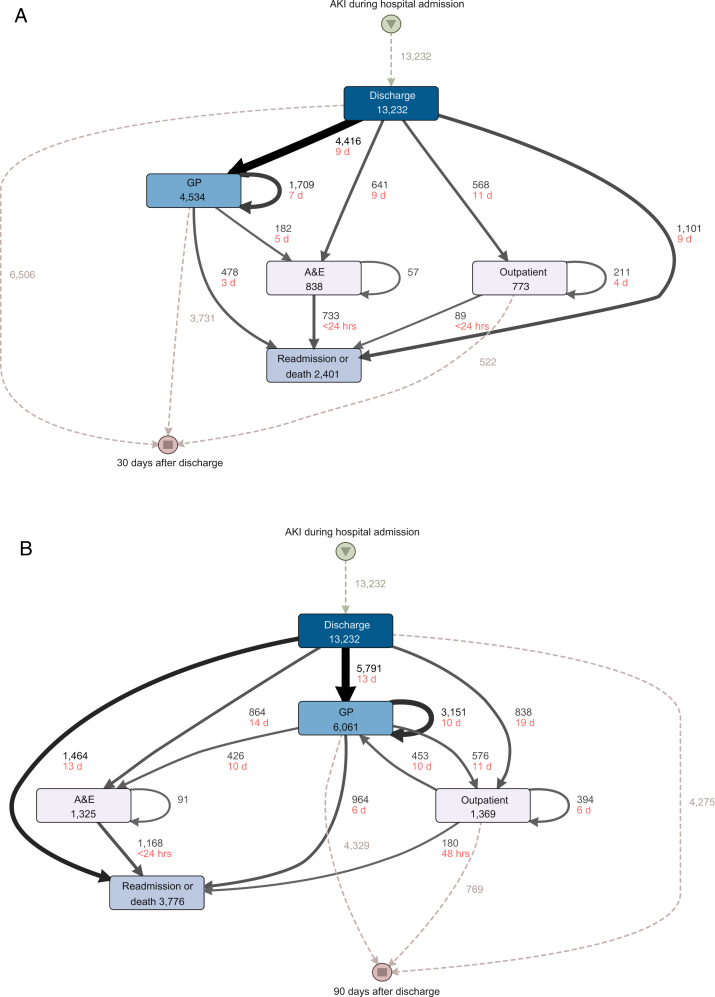
Care processes over the first (A) 30 and (B) 90 days after discharge, following hospital admission with AKI. The figure shows the flow of people from hospital discharge through care events including the accident and emergency department (A&E); primary care general practice (GP); outpatient specialty clinics (outpatient), and hospital readmission or death. Dashed lines represent cohort entry following discharge and exit after 30 and 90 days have elapsed, respectively. Directed arrows are the most common paths between events, weighted by number of people. Numbers beside the arrows between boxes represent the movement of people, and the median days (d) and hours (hrs) between events underneath.

Overall, of 105,461 people discharged from a hospital admission in Grampian between 2011-2014, 9,220 (9%) people died or were readmitted within 90 days, of which 3,776 (41%) were recovering after an AKI episode. Figure 3 focuses on the care processes of those with AKI over the 30 days (Fig 3A) and 90 days (Fig 3b) after leaving the hospital. Of 13,232 people discharged after AKI, 3,776 (29%) were readmitted or died within 90 days (Fig 3B). Most of the kidney function monitoring that occurred within 30 and 90 days was conducted in primary care. A further 1,369 of 13,232 (10%) were assessed in an outpatient specialty clinic within 90 days and 1,325 of 13,232 (10%) attended an emergency department. A lack of any post-AKI monitoring between discharge and readmission was evident in 1,101 of 2,401 (42%) deaths/readmissions within 30 days (Fig 3A), and 1,464 of 3,776 (39%) deaths or readmissions within 90 days (Fig 3B) after AKI. The median times to these unmonitored poor outcomes were 9 and 13 days.

## Discussion

Poor outcomes after hospitalization with AKI are increasingly recognized, but how to perform optimal follow-up care is uncertain. In this validation study of 2 risk prediction models, both performed well in predicting individualized risks of death or readmission (for all hospital survivors) and risks of progression to CKD G4-G5 (for AKI survivors) and were superior to alternative strategies for targeting people follow-up, including indiscriminate follow-up of all people with AKI, or those with severe AKI. We also showed that while poor outcomes are particularly common after AKI, many people receive little or no postdischarge monitoring, which suggests opportunities exist to develop follow-up interventions to improve postdischarge care.

While the current KDIGO AKI guideline suggests a post-AKI clinical reassessment for all patients, emerging AKI return clinics are often limited to those with severe AKI episodes. Our findings do not support this approach. Among all people leaving hospital, either a model-guided approach or follow-up of all people were the most beneficial approaches. By contrast, a strategy of targeted follow-up based on AKI severity was not only inferior, but also had potential for net harm at clinically relevant risk thresholds for death or readmission. Targeting care based on recovery of kidney function at discharge was also inferior to a model-based approach for death or readmission avoidance and at most risk thresholds for progression to CKD G4-G5. Thus, our analyses suggest that incorporating risk prediction models into postdischarge and post-AKI care planning could improve follow-up planning over alternative “one size fits all” approaches that are currently being promoted in guidelines and existing clinical practices.

Through process mining, our analysis shows that almost half of those readmitted after AKI had received no intervening monitoring over a median 2 weeks prior to their readmission, with recurrent presentations frequently occurring through emergency departments. A prior AKI episode had also occurred in half of all people presenting to hospital as an early readmission. This reinforces the likelihood of there being missed opportunities to avoid readmissions, and the need for early monitoring (within 2 weeks) for many people rather than waiting until 90 days, which may be too late. Thus, clinical decision support systems for post-AKI care should be evaluated to ensure that care is appropriately timed as well as being targeted to those with greatest need.^2^^,^^9^^,^^26^

Strengths of this analysis include the population-based nature of our data sources, with capture of blood tests from all clinical locations in Grampian, to ensure that all people with AKI in the region can be identified and followed throughout a complete illness course. Our assessment of net benefit using decision curve analysis is another strength, enabling us to evaluate the clinical consequences of decisions made using a model and to compare whether decisions based on models will do more harm than good when compared with alternate decision-making strategies.

There are further methodological considerations to highlight. Prior to updating, both models overpredicted risks. This confirms the need for updating of risk models for clinical use in new populations and over time.^27^ While death, readmissions, and CKD are important outcomes, it is also important to recognize that nephrologists and other clinicians have a broader role in caring for people with kidney disease, who may also have other comorbidities that impact hospitalization, and diverse social backgrounds and health needs that cannot be fully accounted for in a risk tool. Moreover, even if an event is correctly predicted, this does not mean that follow up or an intervention will be beneficial. For instance, some people may be receiving palliative care, and others will have reasons for poor outcomes that are unavoidable. Other factors we have not considered include barriers to health care access: health literacy, education, cognitive function, physical disability, geographic distance to clinics, and affordability to travel to follow-up appointments.

Accurate prediction of postdischarge outcomes alone does not make a model useful for clinical practice. This analysis goes further by showing that model-guided decisions are superior to alternative approaches for predicting poor health outcomes after hospitalization, but nevertheless, those with the highest risks of poor outcomes may not have the most to gain from an intervention. Without a trial, our analysis cannot confirm which people leaving hospital would have readmissions prevented by an early nonspecialist review, nor can it confirm which AKI survivors would have CKD G4 complications prevented by ongoing kidney care. We also recognize that the health needs and reasons for readmission of people with AKI may require a different approach to follow-up intervention to those without AKI. For instance, clinical fluid assessment to prevent incipient pulmonary edema will be particularly important for those who have had medications stopped.

In conclusion, we have shown that risk prediction models for death or readmission and CKD have the potential to assist in prioritizing people who have had AKI within follow-up care planning and may be superior to alternative strategies such as prioritizing on AKI severity or kidney recovery alone. Further, many people with poor outcomes after AKI receive little or no postdischarge monitoring. A necessary next step is to design and trial risk model–assisted decisions that triage people into appropriate models of postdischarge care that provide the most appropriate level of specialist/nonspecialist input at the most appropriate timepoint.
