# Supplementary material for: Validation of Risk Prediction Models to Inform Clinical Decisions After Acute Kidney Injury
Source: Am J Kidney Dis. 2021 Jul;78(1):28–37. doi: 10.1053/j.ajkd.2020.12.008 (PMC8234511; doi:10.1053/j.ajkd.2020.12.008)
Supplement: Supplementary File (PDF) — Figure S1; Tables S1-S2. [file mmc1.pdf]

Figure S1. Decision curve analysis for net benefit of the Aberdeen readmissions model compared with alternative decision strategies, restricting the cohort to only AKI survivors rather than all hospital survivors.

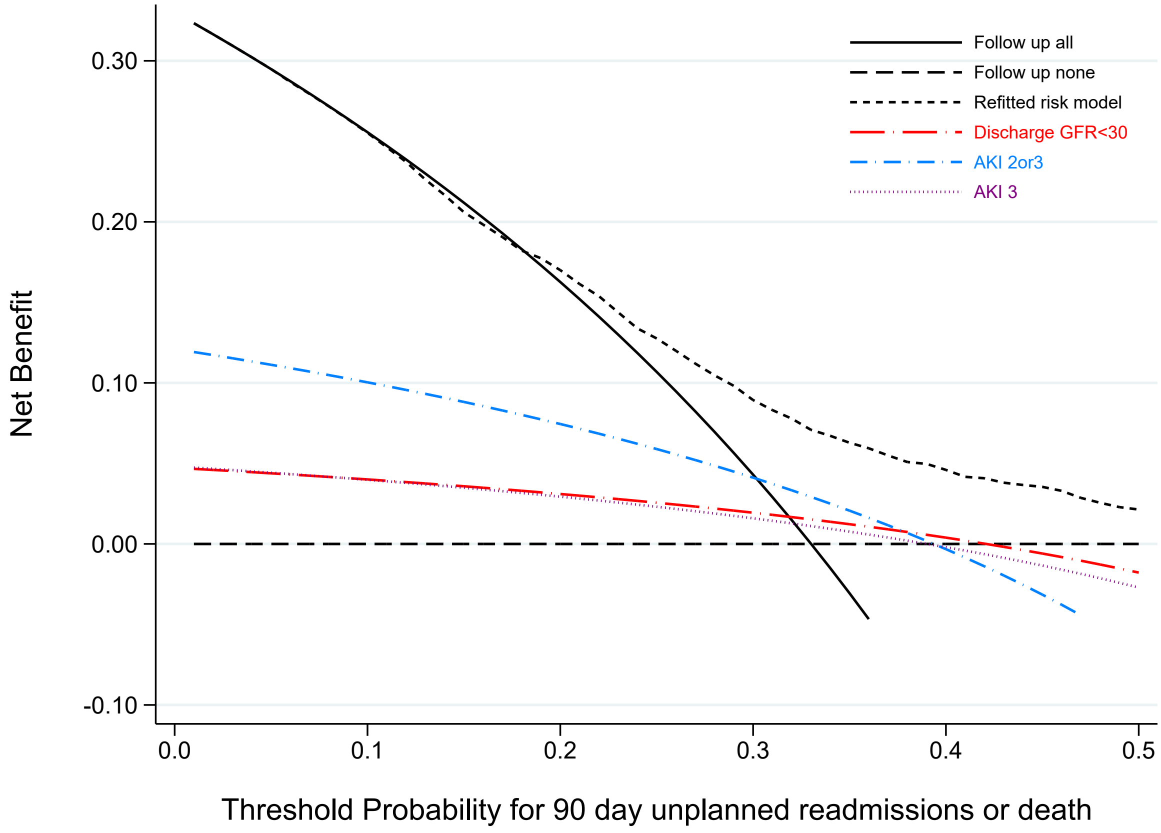

Table S1. Analysis of net benefit at relevant decision threshold for readmissions models

Readmissions model derivation Grampian 2003	Test+ outcome+	Test - outcome +	Test + outcome -	Test - outcome -	% Test positive	% Correct	Net benefit
Original model (30% threshold)	962	2103	1345	12043	14.02	79.04	0.0234
Treat all (30% threshold)	3065	0	13388	0	100.00	18.63	-0.1624
Discharge GFR<30 (yes/no)	228	2837	435	12953	4.03	80.11	0.0025
AKI of any stage (yes/no)	911	2154	1712	11676	15.94	76.50	0.0108
AKI stage 2 or 3 (yes/no)	383	2682	522	12866	5.50	80.53	0.0097
AKI stage 3 (yes/no)	150	2915	181	13207	2.01	81.18	0.0044

Readmissions model external validation Grampian 2012	Test+ outcome+	Test - outcome +	Test + outcome -	Test - outcome -	% Test positive	% Correct	Net benefit
Original model (30% threshold)	1612	1315	3771	19877	20.26	80.86	-0.0002
Recalibrated model (30% threshold)	765	2162	1073	22575	6.92	87.83	0.0115
Refitted model (30% threshold)	902	2025	1264	22384	8.15	87.62	0.0136
Treat all (30% threshold)	2927	0	23648	0	100.00	11.01	-0.2712
Discharge GFR<30 (yes/no)	208	2719	489	23159	2.62	87.93	-0.0001
AKI of any stage (yes/no)	1096	1831	2224	21424	12.49	84.74	0.0054
AKI stage 2 or 3 (yes/no)	402	2525	619	23029	3.84	88.17	0.0051
AKI stage 3 (yes/no)	160	2767	250	23398	1.54	88.65	0.0020

Abbreviations: AKI, acute kidney injury; eGFR, estimated glomerular filtration rate; CKD G4, chronic kidney disease stage G4
Note: Net benefit is calculated from true positives minus false positives with false positives weighted by the decision threshold

Table S2. Analysis of net benefit at relevant decision thresholds for CKD G4/5 models

CKD G4/5 model derivation Alberta	Test+ outcome+	Test - outcome +	Test + outcome -	Test - outcome -	% Test positive	% Correct	Net benefit
Original model (1% threshold)	249	23	4464	5237	47.26	55.01	0.0204
Treat all (1% threshold)	272	0	9701	0	100.00	2.73	0.0174
Discharge GFR<30 (yes/no)	112	160	393	9308	5.06	94.46	0.0108
AKI stage 2 or 3 (yes/no)	136	136	2151	7550	22.93	77.07	0.0115
AKI stage 3 (yes/no)	91	181	839	8862	9.33	89.77	0.0083
Original model (10% threshold)	129	143	522	9179	6.53	93.33	0.0071
Treat all (10% threshold)	272	0	9701	0	100.00	2.73	-0.0808
Discharge GFR<30 (yes/no)	112	160	393	9308	5.06	94.46	0.0069
AKI stage 2 or 3 (yes/no)	136	136	2151	7550	22.93	77.07	-0.0103
AKI stage 3 (yes/no)	91	181	839	8862	9.33	89.77	-0.0002

CKD G4/5 external validation Grampian 2011-2013	Test+ outcome+	Test - outcome +	Test + outcome -	Test - outcome -	% Test positive	% Correct	Net benefit
Original model (1% threshold)	124	16	3745	5497	41.24	59.91	0.0092
Recalibrated model (1% threshold)	122	18	3049	6193	33.80	67.31	0.0097
Refitted model (1% threshold)	124	16	3107	6135	34.44	66.71	0.0099
Treat all (1% threshold)	140	0	9242	0	100.00	1.49	0.0050
Discharge GFR<30 (yes/no)	37	103	255	8987	3.11	96.18	0.0037
AKI stage 2 or 3 (yes/no)	59	81	1962	7280	21.54	78.22	0.0042
AKI stage 3 (yes/no)	33	107	749	8493	8.34	90.88	0.0027
Original model (10% threshold)	51	89	352	8890	4.30	95.30	0.0013
Recalibrated model (10% threshold)	31	109	190	9052	2.36	96.81	0.0011
Refitted model (10% threshold)	29	111	168	9074	2.10	97.03	0.0011
Treat all (10% threshold)	140	0	9242	0	100.00	1.49	-0.0945
Discharge eGFR<30 (yes/no)	37	103	255	8987	3.11	96.18	0.0009
AKI stage 2 or 3 (yes/no)	59	81	1962	7280	21.54	78.22	-0.0169
AKI stage 3 (yes/no)	33	107	749	8493	8.34	90.88	-0.0054

Abbreviations: AKI, acute kidney injury; eGFR, estimated glomerular filtration rate; CKD G4, chronic kidney disease stage G4
Note: Net benefit is calculated from true positives minus false positives with false positives weighted by the decision threshold
